# Thalamic GABA+ levels are negatively associated with neuropsychiatric symptoms in patients with insomnia

**DOI:** 10.3389/fnhum.2026.1750271

**Published:** 2026-02-05

**Authors:** Mingyuan Dai, Huande Hong, Yumeng Mao, Rui Wang, Yanlong Jia, Dongyuan Xu, Gen Yan

**Affiliations:** 1 Key Laboratory of Cellular Function and Pharmacology of Jilin Province, Yanbian University, Yanji, China; 2 Department of Radiology, The Second Affiliated Hospital of Xiamen Medical College, Xiamen, China; 3 Department of Radiology, Xiamen Fifth Hospital, Xiamen, China; 4 Department of Radiology, Second Affiliated Hospital, Shantou University Medical College, Shantou, Guangdong, China; 5 Department of Radiology, Xiangyang Central Hospital, Affiliated Hospital of Hubei University of Arts and Science, Xiangyang, Hubei, China

**Keywords:** glycerophosphocholine, insomnia, magnetic resonance spectroscopy, sleep disorders, thalamus, *γ*-aminobutyric acid

## Abstract

**Objective:**

Insomnia is the most common type of sleep disorder; however, the neurobiological causes and correlates of hyperarousal in insomnia remain to be fully determined, and the levels of cerebral metabolites in patients with insomnia remain unclear. This study aimed to quantify changes in cerebral metabolite levels in patients with insomnia and to explore their relationship with fatigue, anxiety, and subjective sleepiness.

**Methods:**

Twenty unmedicated patients with insomnia disorder and 21 age- and sex-matched healthy volunteers were included. The concentrations of metabolites including *γ*-aminobutyric acid (GABA+), glutamate (Glu), glycerophosphocholine (GPC), creatine (Cr), and phosphocreatine (PCr) were obtained by magnetic resonance spectroscopy, and the differences in metabolites between the two groups were compared. Sleep quality, sleepiness, anxiety, and fatigue were assessed using the Pittsburgh Sleep Quality Index (PSQI), Karolinska Sleepiness Scale (KSS), Beck Anxiety Inventory (BAI), and Fatigue Severity Scale (FSS), respectively. Correlations between the changes in GABA+, Glu, and GPC levels and the PSQI, KSS, FSS, and BAI scores were evaluated in patients with insomnia.

**Results:**

GABA+ levels were significantly lower in patients with insomnia than in healthy controls (*p* = 0.027), whereas GPC and Cr+PCr levels were significantly higher (*p* < 0.001 and *p* = 0.003, respectively). However, Glu levels were comparable between the groups (*p* = 0.962). Furthermore, GABA+ levels were significantly negatively correlated with FSS (r = −0.656, *p* = 0.003) and BAI (r = −0.467, *p* = 0.038) scores; a trend-level negative association with KSS was also observed (r = −0.419, *p* = 0.066).

**Conclusion:**

Our results revealed alterations in the levels of GABA+ and GPC in the thalamus of patients with insomnia. These findings provide objective neurochemical evidence for the pathophysiological mechanisms of insomnia.

## 1 Introduction

Adequate sleep quality is crucial for physical health, and insufficient sleep quality has detrimental health effects (Spytska, 2024). The National Sleep Foundation recommends that adults achieve 7–9 h of sleep per night (Wickwire et al., 2019); similarly, the American Heart Association and the Centers for Disease Control and Prevention advocate for adults to get at least 7 h of sleep per night to promote optimal health and reduce the risk of disease (St-Onge et al., 2016). Given the critical role of sleep quality in overall well-being, sleep research has gained attention in recent decades. Insomnia is the most common type of sleep disorder. According to the International Classification of Sleep Disorders, Third Edition (ICSD-3), the diagnosis of insomnia must satisfy three criteria: persistent sleep difficulties, adequate opportunity for sleep, and associated daytime functional impairment (Thorpy, 2017). Insomnia is identified through ongoing, self-reported sleep disturbances that interfere with everyday functioning. Research has indicated that people with insomnia face an increased risk of developing and sustaining various psychiatric conditions, especially depression and anxiety (Hertenstein et al., 2019). Furthermore, insomnia elevates the risk of cardiovascular disease (Khan and Aouad, 2022), obesity, and diabetes (Duan et al., 2023).

In the fast-paced lifestyle of modern society, increasing levels of stress have made insomnia a widespread public health issue. In support, the China Family Panel Studies’ nationally representative statistics revealed that adults have seen their average sleep duration slightly decline from 2010 to 2016. However, from 2016 to 2018, the average sleep duration remained relatively stable (Chu et al., 2023). Poor sleep quality is a persistent problem for many people. As well as causing fatigue, tiredness, and reduced attention and motivation, insomnia may contribute to anxiety and depression, impaired social and occupational functioning, and errors or accidents (Chung et al., 2015). Untreated insomnia increases healthcare costs; patients treated for sleep disorders show higher healthcare utilization and costs compared with those without sleep disorders (Wickwire et al., 2019).

Although sleep is a fundamental physiological function, its mechanisms are quite complex (Bishir et al., 2020). As such, sleep remains a highly active area of research. Studies have shown that multiple brain regions are involved in promoting sleep, among which the thalamus plays a crucial role in sleep regulation (Hale et al., 2016). The thalamus is a key brain region involved in the pathophysiology of the sleep–wake cycle, hyperarousal, emotion, and restorative autonomic and endocrine processes (Huang et al., 2022). The thalamus plays a key role in sleep through thalamocortical circuits, and by regulating cortical excitability and the transmission of sensory information, it influences the onset and maintenance of sleep. Therefore, in the present study, the thalamus was selected as the region of interest, given its dysfunction may be related to the neurobiological mechanisms of insomnia. Multiple studies have shown a significantly reduced grey matter volume in the thalamus of individuals experiencing chronic total sleep deprivation (Liu et al., 2022; Li et al., 2021). However, the specific brain mechanisms underlying insomnia remain unclear, with the hyperarousal theory providing a strong basis for its pathophysiology (Dressle and Riemann, 2023). Therefore, in the current study, spectroscopy of the right thalamus was performed to quantify cerebral metabolite levels in patients with insomnia and, subsequently, to evaluate the relationship of metabolite levels with anxiety, fatigue, and tiredness.

Navigating the intricacies of pinpointing brain regions crucial for cognition and learning without resorting to intrusive or radioactive means is challenging. However, advances in neuroimaging technology have supported the utilization of magnetic resonance spectroscopy (MRS) as a go-to tool. MRS offers a reliable, non-invasive method for assessing the metabolic activity of the brain (Faghihi et al., 2017). The emergence of MRS has enabled accurate *in vivo* quantification of brain neurometabolites, such as *γ*-aminobutyric acid (GABA), glutamate (Glu), and the composite measure of Glu and glutamine (Gln), known as Glx (Puts and Edden, 2012). Nonetheless, conventional MRS techniques often encounter significant interference from water, lipids, and other macromolecular signals when detecting metabolites, such as GABA. By optimizing specific echo time (TE) and editing pulses, the MEGA-PRESS sequence selectively inverts the J-coupled resonances of target metabolites. Through subtraction of ON and OFF spectra, it retains only the signals of the intended metabolites, thereby effectively suppressing background interference (An et al., 2018). A major advantage of MEGA-PRESS lies in its enhanced sensitivity for detecting low-concentration metabolites. Utilizing J-difference editing, this technique can extract GABA signals from overlapping strong background signals, significantly improving both specificity and sensitivity in detection (Gu et al., 2017).

GABA and Glu are key neurotransmitters, playing vital roles in the central nervous system and in maintaining the physiological functions of the brain. Benzodiazepines, a class of central nervous system depressants, enhance the inhibitory effects of GABA in the central nervous system, thus aiding restful sleep (Bertisch et al., 2014). Reports have reported reduced GABA+ levels in the occipital cortex and anterior cingulate cortex of patients with primary insomnia (Winkelman et al., 2008). Therefore, cerebral metabolite alterations in patients with insomnia may serve as a target for the treatment or prevention of insomnia symptoms. Moreover, a study by Kakeda et al. (2011) showed significant differences between morning and evening measurements of GABA+ levels in the frontal lobe. Therefore, in the present study, we uniformly controlled the timing of MRS scans to a specific window (18:30–19:30).

Owing to the current knowledge gaps regarding the neurobiological causes and correlates of hyperarousal in insomnia, this study aimed to examine the relationship between GABA+ levels and symptoms of fatigue, anxiety, and tiredness. We hypothesized that GABA+ levels would be reduced in patients with insomnia compared with healthy individuals.

Recent systematic reviews and large-cohort studies have provided further evidence for the neurobiological basis of insomnia and its psychiatric comorbidities (Aquino et al., 2024; Palagini et al., 2023). Additionally, advances in reproducible MRS analytic strategies have improved the reliability of neurometabolite quantification in clinical populations (Peek et al., 2023).

## 2 Materials and methods

### 2.1 Participants

A total of 41 participants were recruited, including 20 patients with insomnia and 21 age- and sex-matched healthy controls. The patients with insomnia (aged 19–39 years) were diagnosed according to the ICSD-3 criteria at the Department of Neurology, Second Affiliated Hospital of Xiamen Medical College, while the healthy controls (aged 20–40 years) had no history of sleep disorders. For a diagnosis of insomnia, the ICSD-3 criteria require the presence of persistent sleep difficulties for > 3 months, difficulty falling asleep (taking > 30 min to fall asleep while in bed at night), and associated daytime functional impairment. All participants were newly diagnosed cases, were not taking any medication for insomnia symptoms, and had been experiencing symptoms for an average of 1.5 years. Each participant provided written informed consent. Before the MRS scans, all participants were fully informed of the procedure, its objectives, and its potential clinical value. To rule out any issues with the peripheral or central nervous systems, all participants underwent a detailed neurological evaluation by a neurologist with more than a decade of experience.

The participant exclusion criteria were as follows: neurological, endocrine, or psychiatric disorders; conventional brain abnormalities (e.g., tumors, hemorrhage, infarction), alcohol or substance abuse; and contraindications for magnetic resonance imaging (MRI) (e.g., metal implants, pacemakers, neurostimulators, body piercings, or claustrophobia). All participants were required to refrain from using any sleep quality-improving medications prior to the metabolite measurement and scale assessments, and to avoid consuming caffeine-containing substances (including coffee, tea, and energy drinks) for at least 24 h beforehand.

Prior to MRS imaging, all healthy control participants reported good sleep quality and regular sleep habits. To assess the degree of daytime tiredness, fatigue, anxiety, and sleep quality, both the patients with insomnia and healthy controls completed the Pittsburgh Sleep Quality Index (PSQI), Fatigue Severity Scale (FSS), Beck Anxiety Inventory (BAI), and Karolinska Sleepiness Scale (KSS) questionnaires.

### 2.2 Conventional MRI data acquisition

All MRI and MRS examinations were performed on a clinical 3.0-T MRI scanner (Discovery MR 750w, GE Healthcare, Milwaukee, WI, United States) equipped with a 24-channel head coil. Prior to the MRS examination, conventional MRI scans were performed to rule out intracranial lesions. The captured images and scanning parameters were as follows: axial T2-weighted fast spin-echo images (repetition time [TR] = 3,500 ms, echo time [TE] = 100 ms, number of excitations [NEX] = 2, field of view [FOV] = 240 × 240 mm^2^, slice thickness = 5 mm, acquisition time = 2 min 6 s); coronal T2-weighted fluid-attenuated inversion recovery (T2 FLAIR) images (TR = 9,000 ms, TE = 145 ms, NEX = 1, FOV = 240 × 240 mm^2^, slice thickness = 5 mm, acquisition time = 2 min 52 s); and sagittal T2 FLAIR images (TR = 9,000 ms, TE = 145 ms, NEX = 1, FOV = 240 × 240 mm^2^, slice thickness = 5 mm, acquisition time = 1 min 57 s).

### 2.3 Conventional MRS and MEGA-PRESS image acquisition

**Figure 1:**
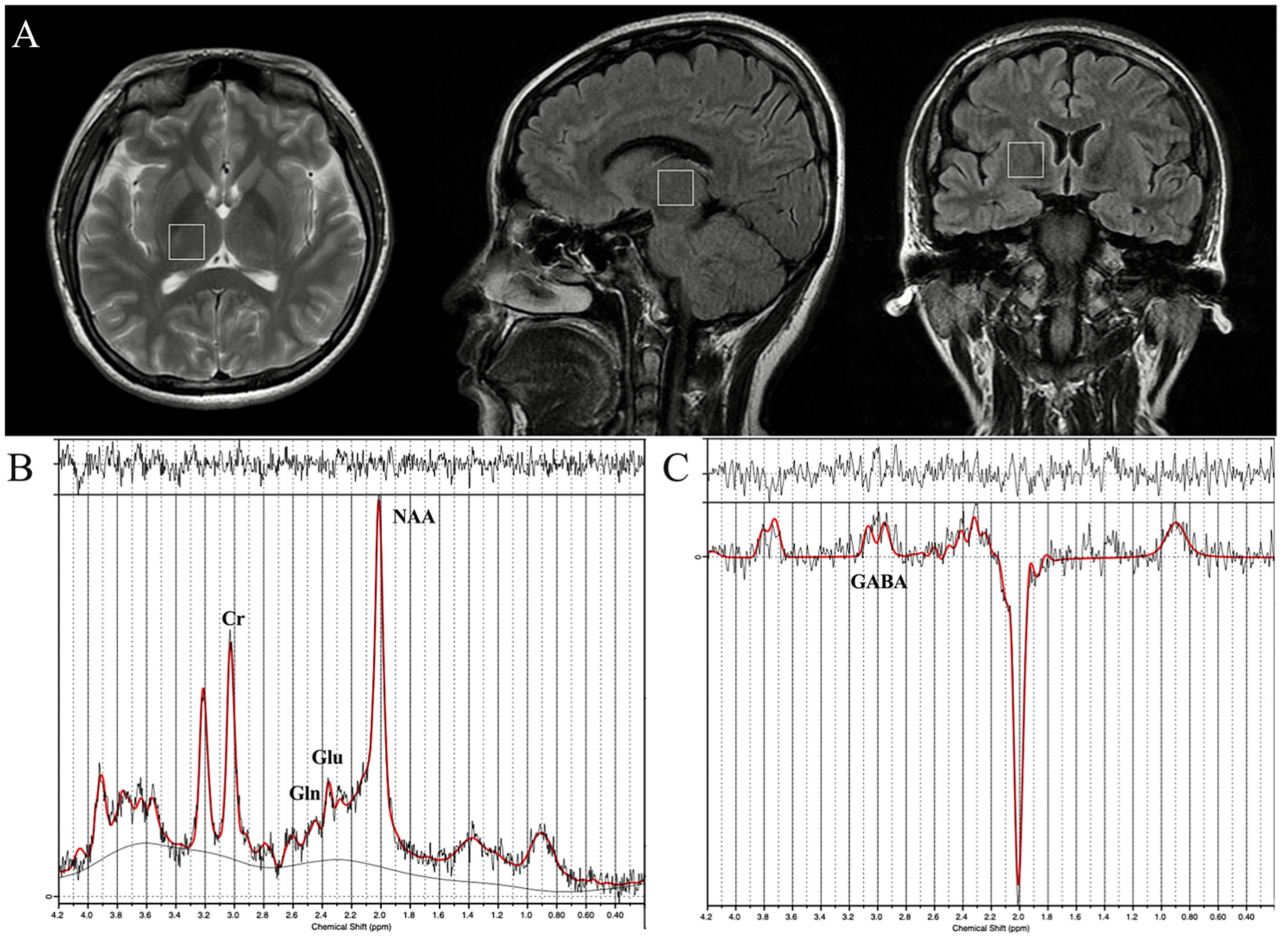
**(A)** Volume of interest in the right thalamus on sagittal, axial, and coronal images; **(B)** PRESS spectrum; **(C)** GABA-edited MEGA-PRESS difference spectrum. Cr, creatine; NAA, N-acetylaspartate; Gln, glutamine; Glu, glutamate; GABA, *γ*-aminobutyric acid.

To avoid potential interference from the skull, sinus structures, and cerebrospinal fluid during scanning, the voxel size was optimized, and a volume of interest (VOI) measuring 20 × 20 × 20 mm^3^ in the right thalamus was selected (Figure 1A). The MRS scanning parameters were as follows: TR = 3,000 ms, TE = 35 ms; number of signal averages = 128; acquisition time = 7 min 36 s. For Mescher–Garwood Point RESolved Spectroscopy (MEGA-PRESS), the scanning parameters were as follows: VOI = 20 × 20 × 20 mm^3^; TR = 1,800 ms, TE = 68 ms; spectral width = 2,000 Hz; data points = 2,048; number of signal averages = 160; acquisition time = 10 min 19 s; total scan time ≤20 min. Editing pulses were applied at “on” frequency (1.7 ppm; editing frequency: −356 Hz) and “off” frequency (7.7 ppm; editing frequency: 356 Hz); the editing pulse shape was set to 12.

The right thalamus was selected based on previous studies suggesting lateralized thalamic involvement in sleep regulation (Aquino et al., 2024; Van Someren, 2021) and to maintain consistency with prior MRS investigations in sleep disorders. Additionally, focusing on a single hemisphere minimized scan time while ensuring adequate spectral quality.

#### 2.3.1 Spectral quality control

Spectra were excluded if the Cramér-Rao Lower Bound (CRLB) for GABA+ exceeded 20% or if the signal-to-noise ratio (SNR) was below 10. Mean spectral quality metrics were as follows: SNR = 18.2 ± 3.4 (insomnia group) vs. 19.1 ± 2.8 (controls); mean full width at half maximum (FWHM) = 0.065 ± 0.012 ppm; frequency drift < 0.5 ppm across acquisition. No participants were excluded based on these quality control criteria. It should be noted that “GABA+” refers to GABA plus co-edited macromolecular contributions at 3 ppm, as is standard for MEGA-PRESS without macromolecule suppression (Peek et al., 2023). The macromolecular component is estimated to contribute approximately 40–60% of the total GABA+ signal.

### 2.4 Scale surveys

The KSS was used to evaluate participants’ subjective tiredness. This scale quantifies the instantaneous alertness/sleepiness state through a 9-point rating system, with scores ranging from 1 (extremely alert) to 9 (extremely sleepy) (Miley et al., 2016).

The BAI was used to assess the severity of participants’ anxiety symptoms and to distinguish anxiety from depressive symptoms. The BAI consists of 21 items, each describing a specific anxiety symptom. The total score ranges from 0 to 63, with scores of 0–7 falling within the normal range, 8–15 indicating possible mild anxiety, 16–25 suggesting moderate anxiety, and 26–63 indicating severe anxiety (Carney et al., 2011).

The FSS is a widely used self-assessment tool designed to evaluate an individual’s subjective perception of fatigue and its impact on daily functioning (Krupp et al., 2020). It assesses the state of fatigue over the past 2 weeks, with higher scores indicating greater severity, frequency, and impact of fatigue on daily life, significantly affecting daily functioning.

### 2.5 MRS data analysis

The Linear Combination Model (LCModel), as a key method for MRS data analysis, aims to quantify the concentrations of various metabolites from complex spectral signals. Quantification of the detected cerebral metabolites was performed using LCModel (version 6.3-1R) spectral analysis software. Quantification was based on the linear combination model and simulated basis sets (including basis spectra for Gln, Glu, N-acetylaspartate, and N-acetylaspartylglutamate) (Figures 1B,C). Water signal-referenced metabolite concentrations were calculated from the edited spectra. Due to the influence of J-coupling effects and T2 attenuation in the MEGA-PRESS sequence, among other factors, the obtained absolute values may contain unknown scaling factors and are not absolute quantitative values.

### 2.6 Statistical analysis

IBM SPSS Statistics IBM SPSS Statistics (version 27.0; IBM Corporation, New York, NY, United States) and GraphPad Prism (version 10.3.1; GraphPad Software, LLC, San Diego, CA, United States) were used for data analysis and visualization. Statistical analyses of demographic characteristics, intracerebral metabolite concentrations, and ESS, FSS, and BAI scores were performed using SPSS, while statistical graphs were generated using GraphPad Prism. The normality of continuous variables in both the overall cohort and subgroups was first assessed using the one-sample Kolmogorov–Smirnov (K-S) test. Metabolite data conforming to a normal distribution were compared between the healthy control and insomnia patient groups using independent samples t-tests and are expressed as mean ± standard deviation (SD). For non-normally distributed data, between-group comparisons were conducted using the Mann–Whitney U test, with results presented as median (interquartile range). The significance level was set at *p* < 0.05. To control for the effects of covariates such as age and sex, analysis of covariance (ANCOVA) was employed when the dependent variables satisfied normality assumptions, while Quade’s ANCOVA was applied for non-normally distributed data. For data visualization purposes, the residual method was utilized to obtain metabolite values adjusted for age and sex. Specifically, linear regression was performed with all participants’ metabolite concentrations as the dependent variable and age and sex as independent variables, from which standardized residuals were saved. These residuals represent the metabolite levels after removing the linear influences of the covariates. All subsequent between-group comparisons were graphically represented using GraphPad Prism based on these adjusted residual data. This integrated approach ensures both statistical rigor in hypothesis testing and intuitive visualization of the true intergroup differences after covariate adjustment.

Primary and Secondary Outcomes: Thalamic GABA+ was designated as the primary metabolite outcome, with FSS and BAI scores as primary symptom endpoints. Other metabolites (GPC, Glu, Cr + PCr) and the KSS correlation were considered secondary/exploratory outcomes. For multiple comparison correction, the Benjamini-Hochberg false discovery rate (FDR) method was applied to metabolite group comparisons. All reported correlations are partial correlations controlling for age and sex; figures display regression residuals after removing covariate effects.

*Post hoc* Power Analysis: Using G*Power 3.1, we calculated achieved statistical power for our primary findings. For the GABA+ group difference with *n* = 20 and *n* = 21, *α* = 0.05, and the observed effect size (Cohen’s d = 0.72), achieved power was 0.68. For the correlation between GABA+ and FSS (r = −0.656), achieved power was 0.89.

The detailed MRS data analysis pipeline is illustrated in Supplementary Figure S1, and comprehensive spectral quality control metrics for both groups are provided in Supplementary Table S1.

## 3 Results

### 3.1 Participant characteristics

After controlling for age and sex, ANCOVA revealed significantly higher FSS scores in the insomnia group than in the control group [*F*(1, 36) = 150.25, *p* < 0.01] (Table 1). A rank-based ANCOVA performed on the non-normally distributed KSS scores similarly indicated that the insomnia group scored significantly higher than the control group [*F*(1, 37) = 136.33, *p* < 0.001]. Among the male participants, the insomnia group showed significantly higher BAI scores compared with the control group (*p* < 0.001). Furthermore, a marginally significant interaction was observed between group and age [*F*(2, 35) = 2.63, *p* = 0.086], suggesting that the effect of age on anxiety may differ across groups.

**Table 1:** Intergroup differences in demographic and scale scores.

Clinical characteristics	HC group (*n* = 21)	ID group (*n* = 20)	Test statistic	*p*-value
Age (years)	27.19 ± 6.02	28.95 ± 7.54	*t* = −0.83	0.413
Sex (M/F)	7/14	7/13	χ^2^ = 0.01	0.910
FSS	1.74 ± 0.66	4.42 ± 0.65	*F* = 150.25	0.001***
BAI	2.38 ± 2.89	16.85 ± 7.53	*F* = 2.63	0.086
KSS	2.00 (1.00)	7.00 (1.00)	*F* = 136.33	<0.001***
PSQI	3.10 ± 1.20	14.20 ± 2.80	*t* = −15.23	<0.001***

Data are presented as mean ± standard deviation or median (IQR). Significant differences are indicated by asterisks, *** *p* < 0.001. HC, healthy control; ID, insomnia disorder; FFS, Fatigue Severity Scale; KSS, Karolinska Sleepiness Scale; BAI, Beck Anxiety Inventory; IQR, interquartile range.

### 3.2 Thalamic neuro-metabolite differences between patients with insomnia and healthy controls

**Figure 2:**
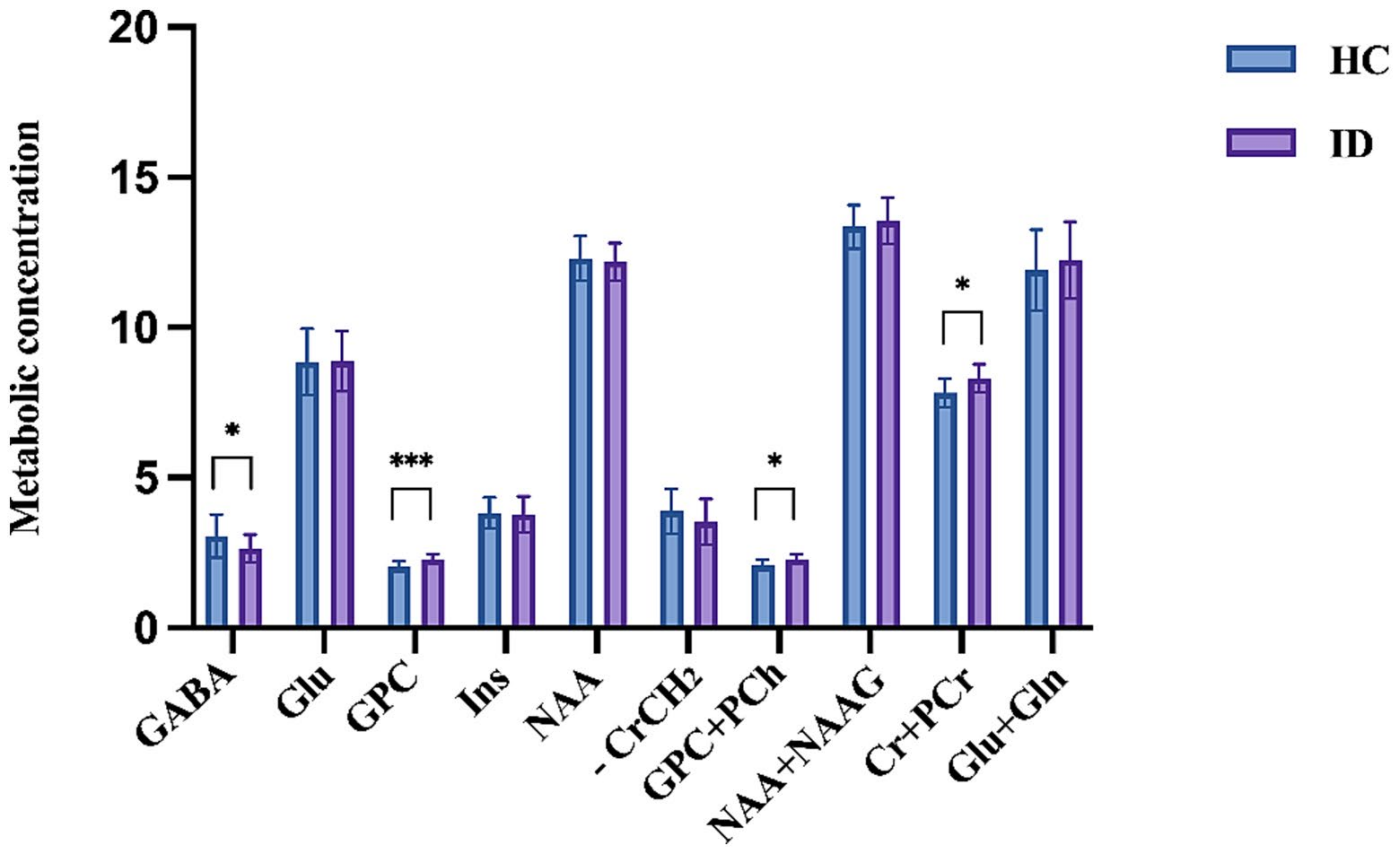
Comparison of metabolites in the thalamus between healthy controls and patients with insomnia. HC, healthy control; ID, insomnia disorder. Significant differences are indicated by asterisks, * *p* < 0.05; ** *p* < 0.01; *** *p* < 0.001.

As shown in Table 2, compared with the healthy control group (mean ± SD: 3.056 ± 0.715), the insomnia group exhibited significantly lower thalamic GABA+ levels (2.623 ± 0.468; *p* = 0.027). In contrast, higher thalamic concentrations were observed in the insomnia group for GPC (2.282 ± 0.166 vs. 2.055 ± 0.172 in controls; *p* < 0.001), GPC + PCh (2.282 ± 0.166 vs. 2.102 ± 0.180; *p* = 0.002), and Cr + PCr (8.294 ± 0.467 vs. 7.832 ± 0.485; *p* = 0.003). Although Glu levels were slightly elevated in the patients with insomnia compared with those in the healthy controls, the difference was not statistically significant (*p* = 0.962). Of note, no alterations in the other measured metabolites were found (Figure 2).

**Table 2:** Comparison of thalamic metabolite levels between groups.

Metabolites	HC group (*n* = 21)	ID group (*n* = 20)	*t*-value	*p*-value
GABA+	3.056 (0.715)	2.623 (0.468)	2.302	0.027*
Glu	8.846 (1.105)	8.862 (1.003)	−0.047	0.962
GPC	2.055 (0.172)	2.282 (0.166)	−4.306	<0.001***
Ins	3.823 (0.521)	3.758 (0.594)	0.374	0.71
NAA	12.300 (0.736)	12.173 (0.627)	0.592	0.557
-CrCH2	3.892 (0.753)	3.508 (0.771)	1.612	0.115
GPC+PCh	2.102 (0.180)	2.282 (0.166)	−3.314	0.002**
NAA+NAAG	13.355 (0.725)	13.526 (0.770)	−0.731	0.469
Cr+PCr	7.832 (0.485)	8.294 (0.467)	−3.109	0.003**
Glu+Gln	11.910 (1.353)	12.226 (1.286)	−0.766	0.448

Data are presented as mean ± standard deviation. GABA, γ-aminobutyric acid; Glu, glutamate; GPC, glycerophosphocholine; PCh, phosphocholine; NAA, N-acetylaspartate; Ins, myo-inositol; Cr, creatine; PCr, phosphocreatine; Gln, glutamine; HC, healthy control; ID, insomnia disorder; SD; standard deviation. Significant differences are indicated by asterisks, *p < 0.05; ** *p* < 0.01;*** *p* < 0.001.

Effect sizes with 95% confidence intervals were calculated for significant metabolite differences: GABA+ group difference: Cohen’s d = 0.72 [95% CI: 0.08–1.35]; GPC group difference: Cohen’s d = 1.35 [95% CI: 0.66–2.02]; Cr + PCr group difference: Cohen’s d = 0.98 [95% CI: 0.32–1.63]. After FDR correction for multiple comparisons across 10 metabolites, GABA+ (pFDR = 0.045), GPC (pFDR = 0.003), and Cr + PCr (pFDR = 0.015) remained statistically significant.

### 3.3 Correlation analysis between cerebral metabolites and subjective scale scores

**Figure 3:**
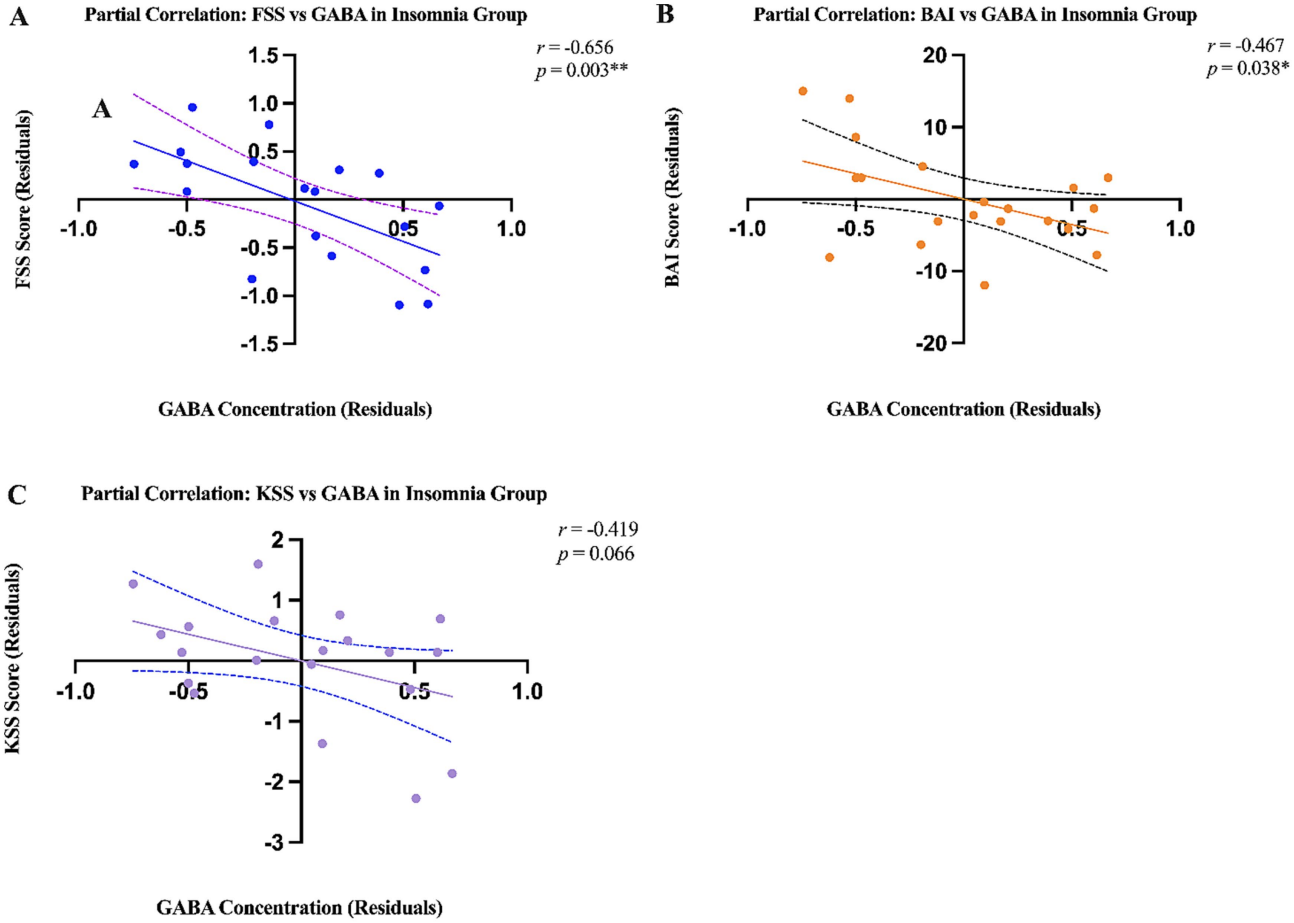
Partial correlation between GABA+ residual values and **(A)** FSS scores, **(B)** BAI scores, and **(C)** KSS scores in patients with insomnia. The scatter plots show regression residuals after controlling for the effects of age and sex. The trend line represents the linear regression fit. Scatter plots display regression residuals of GABA+ concentrations and scale scores after controlling for age and sex. The partial correlation coefficients and 95% confidence intervals are: GABA+–FSS: *r* = −0.656 [95% CI: −0.86 to −0.30], *p* = 0.003; GABA+–BAI: *r* = −0.467 [95% CI: −0.75 to −0.05], *p* = 0.038; GABA+–KSS: *r* = −0.419 [95% CI: −0.71 to 0.03], *p* = 0.066 (trend-level, not statistically significant). FFS, Fatigue Severity Scale; KSS, Karolinska Sleepiness Scale; BAI, Beck Anxiety Inventory; GABA, γ-aminobutyric acid; TH, thalamus. Significant differences are indicated by asterisks, *p < 0.05; ** *p* < 0.01; ^#^*** *p* < 0.001.

To assess the potential links between cerebral metabolites in patients with insomnia and their levels of anxiety, subjective sleepiness, and fatigue, a residual-based regression approach controlling for age and sex was employed, thereby obtaining more precise estimates, despite the lack of significant demographic differences between the groups. Analysis revealed a significant negative correlation between GABA+ levels and FSS scores (*r* = −0.656, *p* = 0.003) (Figure 3A). Furthermore, thalamic GABA+ concentrations were significantly negatively correlated with BAI scores (*r* = −0.467, *p* = 0.038) (Figure 3B), and while a negative trend with the KSS was observed, this did not reach statistical significance (*r* = −0.419, *p* = 0.066) (Figure 3C).

Confidence intervals for correlation coefficients were as follows: GABA+–FSS: r = −0.656 [95% CI: −0.86 to −0.30]; GABA+–BAI: r = −0.467 [95% CI: −0.75 to −0.05]; GABA+–KSS: r = −0.419 [95% CI: −0.71 to 0.03]. The KSS correlation did not reach statistical significance (p = 0.066) and should be interpreted as a trend-level association requiring confirmation in larger samples.

## 4 Discussion

In this study, we identified differences in thalamic metabolite levels between patients with insomnia and healthy controls. Compared with the control group, the insomnia group exhibited lower GABA+ levels, which is consistent with previous observations (Zhang et al., 2022). In contrast, Morgan et al. reported higher occipital GABA+ levels in patients with primary insomnia compared with controls (Morgan et al., 2012; Benson et al., 2020). Furthermore, the present study revealed a negative association of GABA+ levels with anxiety, fatigue, and subjective sleepiness scores. Good sleep quality is crucial for mental health, and compelling evidence indicates that insomnia frequently co-occurs with various psychiatric disorders (Hertenstein et al., 2022). Insomnia symptoms are prevalent in almost all patients with psychiatric conditions. A study by Ren et al. demonstrated that patients with anxiety disorders typically exhibit lower brain GABA+ levels (Ren et al., 2016; Xu et al., 2025). In support, many anxiolytic drugs, such as benzodiazepines, exert their therapeutic effects by enhancing GABAergic activity (Möhler, 2012). Furthermore, a study by Green et al. indicated that GABAergic neurotransmission is closely involved in the regulation of anxiety (Green et al., 2020). In the current study, lower GABA+ levels were associated with anxiety and fatigue.

Methodological Considerations for GABA+ Interpretation: It is important to note that MEGA-PRESS without macromolecule suppression yields “GABA+,” which includes contributions from co-edited macromolecules at 3 ppm (estimated ~40–60% of the signal) (Peek et al., 2023). Therefore, our findings reflect alterations in the composite GABA+ signal rather than pure GABA+ concentrations. While reduced GABA+ is consistent with diminished GABAergic tone, we cannot exclude the possibility that macromolecular changes also contribute to the observed group differences. Future studies using macromolecule-suppressed sequences (e.g., MEGA-SPECIAL) are warranted to isolate pure GABA signals.

Multiple studies have indicated that GABA plays a role in promoting sleep and reducing stress. Alterations in GABA+ levels are closely linked to fatigue, particularly chronic fatigue (Hepsomali et al., 2020; Inotsuka et al., 2021), confirming the critical role of GABA in sleep. Studies have shown that the sleep–wake cycle is closely associated with the dynamic changes in Glu levels in the brain (Weigend et al., 2019). One study found that failure to decrease Glu levels in the medial prefrontal cortex during non-rapid eye movement sleep may contribute to transient insomnia (Yamada et al., 2024). This suggests that insufficient sleep can disrupt the normal metabolic processes of Glu, thereby interfering with sleep maintenance. However, in our present study, no statistically significant difference in thalamic Glu levels was observed between the insomnia and control groups.

The lack of significant glutamate alterations in our study warrants further consideration. In contrast to some previous studies reporting altered glutamate levels in insomnia (Yamada et al., 2024), we found no significant difference in thalamic Glu concentrations between groups. This discrepancy may reflect regional specificity, as prior studies examined prefrontal regions rather than the thalamus (Schiel et al., 2023). Additionally, glutamate alterations may be more pronounced during specific sleep stages or following acute sleep deprivation (Javaheripour et al., 2023), rather than in the chronic insomnia state examined here. The balance between excitatory and inhibitory neurotransmission, rather than absolute Glu levels alone, may be more relevant to insomnia pathophysiology (Van Someren, 2021).

Conversely, GPC and Cr + PCr levels both were higher in the insomnia group than in the control group. A potential association may exist between GPC and insomnia, although no studies to date have directly addressed the relationship between GPC and insomnia. GPC is a choline compound and serves as a precursor to acetylcholine in the brain, playing an important role in nervous system function (Liu et al., 2022). We hypothesize that GPC may indirectly modulate the balance between GABA and Glu by influencing acetylcholine synthesis, thereby affecting sleep. Sleep is regulated by multiple neurotransmitters and metabolic processes, and GPC may indirectly impact sleep quality through its effects on the cholinergic neurotransmitter system (Tsuneki et al., 2016). The cholinergic system plays a key role in sleep regulation. However, further research is needed to elucidate the impact of GPC on sleep.

Revised Interpretation of GPC Findings: While we observed elevated GPC levels in the insomnia group, the lack of significant correlations with symptom measures and the absence of prior studies directly linking GPC to insomnia limit our ability to draw firm conclusions. The elevated GPC may reflect altered membrane phospholipid metabolism or cholinergic activity, but this interpretation remains speculative. Further studies specifically designed to investigate the role of choline-containing compounds in insomnia are needed before mechanistic conclusions can be drawn.

In the present study, the MRS-derived Cr + PCr level reflects the total creatine (tCr) level, as conventional MRS cannot distinguish between Cr and PCr. Both forms play crucial roles in cellular energy metabolism, particularly in the brain and muscles, where they participate in energy buffering and transport through the creatine kinase system (Dworak et al., 2017). A preliminary study on healthy adults indicated that 24-h sleep deprivation significantly increased serum creatine levels (Todorovic et al., 2025), suggesting that insufficient sleep may alter creatine metabolism. Another study using ^31^P-MRS investigated quantitative PCr levels in 30 healthy individuals during wakefulness and nap periods, finding that PCr levels in the left thalamus decreased during wakefulness and increased after napping (Gordji-Nejad et al., 2018). This finding supports the hypothesis that PCr levels are associated with the degree of alertness. Although there are currently no direct data on the relationship between sleep and tCr levels, it may be speculated that sleep influences tCr levels through multiple pathways.

### 4.1 Limitations

Several limitations of the present study should be acknowledged. First, the modest sample size (*n* = 41) limits statistical power, particularly for correlation analyses. The trend-level association between GABA+ and KSS (r = −0.419, *p* = 0.066) should be interpreted with caution and requires confirmation in larger cohorts. *Post hoc* power analysis indicates that detecting correlations of r = 0.40 with 80% power would require approximately 46 participants per group.

Second, tissue-fraction correction (GM/WM/CSF segmentation) was not performed in the present study. Variations in GM/WM/CSF proportions within the thalamic VOI may have contributed to inter-individual variability in metabolite estimates. Future studies should incorporate segmentation-based partial volume correction to improve quantification accuracy.

Third, our focus on the right thalamus precludes assessment of potential hemispheric differences in metabolite alterations. Future bilateral MRS studies are needed to investigate whether similar changes occur in the left thalamus (Aquino et al., 2024).

Fourth, the absence of objective sleep measures such as polysomnography (PSG) or actigraphy limits the interpretability of our findings. While all patients met ICSD-3 diagnostic criteria for insomnia based on clinical evaluation and standardized questionnaires, the lack of objective sleep architecture data precludes assessment of relationships between neurometabolite alterations and specific sleep parameters (e.g., sleep efficiency, wake after sleep onset, slow-wave sleep duration). Future studies incorporating PSG would enable more precise phenotyping.

Fifth, our findings require replication in larger, more diverse cohorts encompassing broader age ranges, varying chronicity levels, and different insomnia subtypes (e.g., sleep-onset vs. sleep-maintenance insomnia) (Palagini et al., 2023).

## 5 Conclusion

In this study, quantitative MRS analysis revealed significantly lower GABA+ levels in the thalamus of patients with insomnia compared with those of HCs, while GPC and Cr + PCr levels were significantly higher; no significant difference was observed in Glu levels. Furthermore, reduced GABA+ levels were directly associated with more severe anxiety symptoms, daytime sleepiness, and fatigue, providing objective neurochemical evidence for the pathophysiological mechanisms of insomnia. However, current research on the direct effects of GPC on sleep remains limited, and the role of GPC in sleep regulation requires further investigation. Future work should explore interventions targeting the GABAergic system to improve clinical symptoms in patients with insomnia. Additionally, large-scale studies are needed to validate the current findings and further evaluate the clinical potential of sleep interventions in the treatment of insomnia.

## Data Availability

The raw data supporting the conclusions of this article will be made available by the authors, without undue reservation.
