# Supplementary material for: Thalamic GABA+ levels are negatively associated with neuropsychiatric symptoms in patients with insomnia
Source: Front Hum Neurosci. 2026 Feb 5;20:1750271. doi: 10.3389/fnhum.2026.1750271 (PMC12916559; doi:10.3389/fnhum.2026.1750271)
Supplement: Supplementary file 1 [file Data_Sheet_1.docx]

**Supplementary Materials**

**Manuscript:** Thalamic GABA+ Levels are Negatively Associated with Neuropsychiatric Symptoms in Patients with Insomnia

**Supplementary Figure S1. MRS Data Analysis Pipeline**

**Analysis Pipeline Schematic:
Step 1: MRI/MRS Data Acquisition**

• 3T Siemens Prisma scanner
• T1-weighted anatomical imaging for VOI localization
• MEGA-PRESS sequence (TE = 68 ms, TR = 2000 ms, 192 averages)

**Step 2: Voxel Placement**

• 20 × 20 × 20 mm³ VOI positioned in right thalamus
• Anatomical landmarks verified by experienced neuroradiologist
• Dual verification for consistency across participants

**Step 3: MRS Preprocessing**

• Frequency and phase correction
• Motion/drift assessment
• Spectral averaging of ON and OFF acquisitions

**Step 4: Metabolite Quantification**

• LCModel version 6.3-1L
• Water-referenced quantification
• Basis set including GABA, Glu, GPC, Cr, PCr, NAA, Ins, and other metabolites

**Step 5: Quality Control**

• SNR threshold: ≥ 10
• CRLB threshold: < 20% for GABA+
• FWHM threshold: < 0.1 ppm
• Frequency drift: < 0.5 ppm

**Step 6: Statistical Analysis**

• Group comparisons: ANCOVA with age and sex as covariates
• Correlation analyses: Partial correlations controlling for age and sex
• Multiple comparison correction: FDR (Benjamini-Hochberg)

**Workflow Diagram:**

┌─────────────────────┐
 │ MRI/MRS Acquisition│
 │ (3T MEGA-PRESS) │
 └──────────┬──────────┘
 │
 ▼
 ┌─────────────────────┐
 │ Voxel Placement │
 │ (Right Thalamus) │
 └──────────┬──────────┘
 │
 ▼
 ┌─────────────────────┐
 │ MRS Preprocessing │
 │ (Frequency/Phase) │
 └──────────┬──────────┘
 │
 ▼
 ┌─────────────────────┐
 │ Quantification │
 │ (LCModel) │
 └──────────┬──────────┘
 │
 ▼
 ┌─────────────────────┐
 │ Quality Control │
 │ (SNR, CRLB, FWHM) │
 └──────────┬──────────┘
 │
 ▼
 ┌─────────────────────┐
 │ Statistical Analysis│
 │ (ANCOVA, FDR) │
 └─────────────────────┘

**Supplementary Table S1**

**MRS Spectral Quality Control Metrics**

| **Parameter** | **Insomnia Group (n=20)** | **Control Group (n=21)** | **p-value** |
| --- | --- | --- | --- |
| SNR | 18.2 ± 3.4 | 19.1 ± 2.8 | 0.352 |
| FWHM (ppm) | 0.065 ± 0.012 | 0.063 ± 0.011 | 0.578 |
| CRLB for GABA+ (%) | 14.3 ± 3.2 | 13.8 ± 2.9 | 0.612 |
| CRLB for Glu (%) | 5.2 ± 1.1 | 5.0 ± 1.0 | 0.545 |
| CRLB for GPC (%) | 3.1 ± 0.8 | 3.0 ± 0.7 | 0.681 |
| Frequency Drift (ppm) | 0.32 ± 0.15 | 0.29 ± 0.12 | 0.467 |

**Note:** Data are presented as mean ± standard deviation. SNR = signal-to-noise ratio; FWHM = full width at half maximum; CRLB = Cramér-Rao Lower Bound. Quality control criteria: SNR ≥ 10, CRLB for GABA+ < 20%, FWHM < 0.1 ppm. No participants were excluded based on these criteria. Between-group comparisons were performed using independent samples t-tests.

**Spectral Quality Assessment:** All acquired spectra met the predefined quality control criteria. The similar spectral quality metrics between groups indicate that any observed metabolite differences are unlikely to be attributable to systematic differences in data quality.

**Note for Figure 1 Revision**

**Suggested updates for Figure 1 (Main Manuscript):**

The current Figure 1 should be updated to include the following panels:

(A) VOI Placement: Current panel showing the 20 × 20 × 20 mm³ voxel location in the right thalamus on sagittal, axial, and coronal T1-weighted images (retain existing).

(B) MEGA-PRESS Acquisition:
 - Top: EDIT-ON spectrum
 - Middle: EDIT-OFF spectrum
 - Bottom: Difference spectrum (ON - OFF)

(C) Spectral Fitting:
 - GABA-edited difference spectrum (blue)
 - LCModel fit (red overlay)
 - Fit residuals (shown below)

(D) Representative Quality Metrics:
 - SNR value
 - FWHM value
 - CRLB values for major metabolites

Note: Representative spectra should be from a participant with SNR and CRLB values close to the group mean to demonstrate typical data quality.
